# Lessons on resilience: Learning to manage complexity

**DOI:** 10.1007/s40037-016-0277-1

**Published:** 2016-06-02

**Authors:** Sayra Cristancho

**Affiliations:** Centre for Education Research & Education, Schulich School of Medicine & Dentistry, Western University, London, ON, Canada

Complexity permeates every aspect of contemporary healthcare, challenging the delivery of medical education [1]. Gormley and Fenwick [2] invite us to reflect on how we can help individual learners to see what’s in front of them when they face complexity. Instead of saying, ‘well, everything is just so chaotic’, the authors offer a practical application of complexity theory that facilitates understanding of* why* things seem to be so chaotic. Gormley and Fenwick’s perspective on complexity, rooted in the field of education, provides the conceptual tools – emergence, self-organization, nested systems – to analyze how things are happening. Since studies on clinical complexity in medical education are growing, in this commentary I would like to offer a complementary perspective to the one put forward by Gormley and Fenwick.

Two special reports published in the Lancet and JAMA have raised concerns about the disconnect between the traditional focus on training individuals as independent practitioners and the expectation that they will perform as collaborative members of healthcare systems [1, 3]. To begin addressing this disconnect, we must consider how we can cultivate a *systems mindset* [3]. While the notion of a systems mindset has been slow to take root in medical education, it is at the very heart of my home discipline of systems engineering. In systems engineering, the ‘big picture’ is everything.

But to see the big picture, systems engineering teaches us that we must focus our attention on the different *dimensions* (e. g. procedural, organizational, personal, social, etc.), and the different *perspectives* (i. e. from multiple actors) that constitute a situation [4–7]. These two notions encapsulate the idea of what it takes to think systemically or to have a systems mindset: depending on the dimension and perspective we consider, a situation will have a particular structure with specific purposes and functions, and will exhibit particular dynamics and a distinct evolution [6]. In other words, different interacting configurations of those dimensions and perspectives – i. e., the parts of a whole – allow for the situation to remain in a continuous state of emergence and self-organization. For example, building rockets reliably did not occur until engineers realized that complex systems become dysfunctional when building parts in isolation – a major issue in the Apollo 13 expedition involved engineers from different specialities struggling to fit a square peg in a round hole!

In systems engineering, ‘resilience’ is a key enabler of emergence and self-organization behaviours during complex situations. While traditionally we have tended to think of ‘resilience’ as a characteristic unique to individuals, systems engineering persuades us to think also of ‘resilience’ as a characteristic of situations. Embracing a systems mindset should help us perceive the degree to which the situation can endure disturbances and allow learning and adaptation – the capacity of the situation to be *resilient* [8, 9].

While ‘resilience’ as a characteristic of situations might be an unusual way of thinking about resilience, it offers three key principles that might be useful when researching complexity in clinical practice [10–12]: (1) treat complex situations as moving targets, (2) think holistically, (3) employ multiple perspectives. While reading Gormley and Fenwick’s paper, I am now realizing that these premises are shared by different scientific disciplines (education and engineering), and more importantly that the field of medical education can benefit from the convergence of these principles across disciplines.

## PRINCIPLE 1: Treat complex situations as moving targets.

Systems engineering holds that complex situations require flexibility in the perspectives we use to analyze them. For each new structure that emerges, each perspective involved in the situation establishes a revised definition of the problematic issue [4, 12].

Education depicts this challenge as ‘diving in to the tumult of dynamic uncertainty’. The strategies used by the students in Gormley’s study – attuning, focusing and orienting – constitute not only coping mechanisms but also tangible indicators that early in training, students (likely in an intuitive manner) understand the need for constantly revising what the situation means at different points in time.

In helping learners develop a ‘systems mindset’ using principle 1, instead of asking, *How do you solve this situation as it stands now?,* we might ask, *How is your interpretation/definition of the situation changing as you attempt to solve it?*

## PRINCIPLE 2: Think holistically.

In systems engineering, attending to how interrelationships among parts are formed helps to identify where patterns of behaviour emerge at a given time. Once those patterns become visible, we begin to appreciate that there is more to situations than what single individuals can see; hence a more comprehensive appreciation of the situation is achieved.

As demonstrated by Gormley and Fenwick, in Education, the sense of paralysis experienced by students when dealing with escalating scenarios suggests a ‘reductionist mindset’. When a complex situation is taken apart it loses its essential properties because the ‘performance of a system depends more on how their parts interact than on how they act independently of each other’ [13].

In helping learners develop a ‘systems mindset’ using principle 2, instead of asking, *What are the details of each element in the situation?*, we might ask, *What are the overall patterns of behaviour that seem to connect the elements in the situation?*

## PRINCIPLE 3: Employ multiple perspectives.

Viewing a complex situation through the lens of multiple individuals is an important practice in systems engineering. Each individual establishes what is within and what is outside the scope of their perspective; the interior contains those parts of the situation that a particular perspective can and must address at a given moment, while the exterior contains the parts of the situation that will be excluded by that particular perspective and likely taken up by another perspective. Multiple perspectives add malleability to how the situation is interpreted, as different perspectives and their intersections bring awareness to different ways of adapting to emerging structures.

‘Recognizing and asserting boundaries’ encapsulate the education framing for employing multiple perspectives. As described by Gormley and Fenwick, educational scenarios that incorporate dynamic complexity require students both to define the boundaries of their professional practice and to put themselves in the other’s shoes. Their clever use of video glasses for data collection might also serve a teaching function, promoting students’ development of this empathic awareness.

In helping learners develop a ‘systems mindset’ using principle 3, instead of asking, *What options do you have at your disposal to deal with this situation?, *we might ask, *Whose perspectives should you consider to help you understand and deal with this situation better?, How does the situation look from others’ perspective (literally and figuratively)?*

## Take home message

The process of applying a systems mindset to a complex situation is a way of bringing to light the different assumptions held by stakeholders or team members about the ways the situation works amidst disturbances. Resilience – the capacity to endure disturbances – is the underlying ability to successfully apply a systems mindset to complex situations. Together with the conceptual tools Gormley and Fenwick recommend, I would suggest that ‘resilience’ also be at the forefront of our attempts to foster a *systems physician mindset* – a mindset that strives for learning and adaptation in the face of unexpected change.
